# Healthcare workers and health care-associated infections: knowledge, attitudes, and behavior in emergency departments in Italy

**DOI:** 10.1186/1471-2334-10-35

**Published:** 2010-02-23

**Authors:** Cristiana Parmeggiani, Rossella Abbate, Paolo Marinelli, Italo F Angelillo

**Affiliations:** 1 Department of Public, Clinical and Preventive Medicine, Second University of Naples, Naples, Italy

## Abstract

**Background:**

This survey assessed knowledge, attitudes, and compliance regarding standard precautions about health care-associated infections (HAIs) and the associated determinants among healthcare workers (HCWs) in emergency departments in Italy.

**Methods:**

An anonymous questionnaire, self-administered by all HCWs in eight randomly selected non-academic acute general public hospitals, comprised questions on demographic and occupational characteristics; knowledge about the risks of acquiring and/or transmitting HAIs from/to a patient and standard precautions; attitudes toward guidelines and risk perceived of acquiring a HAI; practice of standard precautions; and sources of information.

**Results:**

HCWs who know the risk of acquiring Hepatitis C (HCV) and Human Immunodeficiency Virus (HIV) from a patient were in practice from less years, worked fewer hours per week, knew that a HCW can transmit HCV and HIV to a patient, knew that HCV and HIV infections can be serious, and have received information from educational courses and scientific journals. Those who know that gloves, mask, protective eyewear, and hands hygiene after removing gloves are control measures were nurses, provided care to fewer patients, knew that HCWs' hands are vehicle for transmission of nosocomial pathogens, did not know that a HCW can transmit HCV and HIV to a patient, and have received information from educational courses and scientific journals. Being a nurse, knowing that HCWs' hands are vehicle for transmission of nosocomial pathogens, obtaining information from educational courses and scientific journals, and needing information were associated with a higher perceived risk of acquiring a HAI. HCWs who often or always used gloves and performed hands hygiene measures after removing gloves were nurses, provided care to fewer patients, and knew that hands hygiene after removing gloves was a control measure.

**Conclusions:**

HCWs have high knowledge, positive attitudes, but low compliance concerning standard precautions. Nurses had higher knowledge, perceived risk, and appropriate HAIs' control measures than physicians and HCWs answered correctly and used appropriately control measures if have received information from educational courses and scientific journals.

## Background

Health care-associated infections (HAIs) are a serious problem in the healthcare services as they are common causes of illness and mortality among hospitalized patients. Currently, between 5% and 10% of patients admitted to acute care hospitals acquire at least one infection and over the last decades the incidence has increased in both the United States and Europe [1-5]. Several effective evidence-based interventions for reducing the occurrence of HAIs has been proposed, and the Centers for Disease Control and Prevention has developed specific guidelines aimed at preventing the transmission of pathogens within the hospital setting [6]. In Italy, health authorities consider extremely desirable that all healthcare institutions establish and maintain a surveillance system for HAIs [7,8].

Emergency care setting is an area in which the potential risk is most imminent for transmission of HAIs to patients or to those healthcare workers (HCWs) who treat them. Indeed, these HCWs are in the forefront of assisting patients prior a diagnosis, who are critically ill, and with life-threatening conditions. To this end, there has been limited attention paid to investigating knowledge, attitudes, and behavior of HCWs' regarding control policies within this setting [9-12]. Therefore, the objectives of this study were to provide an assessment of the level of knowledge, attitudes, and compliance regarding standard precautions about HAIs among HCWs in emergency departments (EDs) in an area of Italy, and to identify the determinants associated with them. It has been hypothesized that participants more knowledgeable perceive a lower risk of acquiring a HAI from the patients and perform their medical duties with better compliance regarding standard precautions and that HCWs who receive information from educational courses and scientific journals are more likely to be more knowledgeable, to perceive a lower risk, and to perform more appropriate behaviors.

## Methods

Between April 2006 and June 2007 a cross-sectional study was conducted at eight randomly selected non-academic acute general public hospitals in the geographic area of Caserta and Naples (Italy). All 550 HCWs working in the EDs were selected.

The medical director and the ED head of each hospital received a letter with the description of the survey and requesting consent for the HCWs to participate. The medical directors addressed a letter to all HCWs explaining the enrollment and the purpose of the survey, assuring that response was completely voluntary, that information provided would be used solely for fulfilling the research aims, and a self-administered anonymous questionnaire accompanied by an envelope to facilitate its return. Consent to participate was implied by the return of the completed questionnaire.

The questionnaire comprised five categories of questions: (1) demographic and occupational characteristics; (2) knowledge about the risks of acquiring and/or transmitting certain HAIs for/to a patient and standard precautions for prevention; (3) attitudes toward precautionary guidelines and perception of the risk of acquiring HAI; (4) practice of standard precautions; and (5) from which sources they received up-to-date information about HAIs [see Appendix A]. Correct answers to each item were based on a review of the available literature as well as policies and guidelines [6,13].

The content of the questionnaire was validated with interviews and discussions with other experts in the field, and it was modified where necessary. Final questionnaire content, comprehensibility, clarity, and format were developed and validated on input of a volunteer sample of 30 HCWs in a small pilot-test.

The study protocol as well as the questionnaire was approved by Ethical Committee of the Second University of Naples.

### Statistical analysis

Multivariate analysis was carried out using stepwise logistic and linear regression techniques to establish whether the predictor variables were independently associated with the following outcomes of interest: knowledge about the risk for a HCW of acquiring both Hepatitis C (HCV) and Human Immunodeficiency Virus (HIV) infections from a patient (Model 1); knowledge that using standard precautions (gloves, mask, protective eyewear) and hands hygiene after removing gloves are HAIs control measures (Model 2); perceived risk of acquiring a HAI from a patient (Model 3); using often or always gloves when at direct contact with a patient and hands hygiene measures after removing gloves (Model 4). For purposes of analysis, the outcome variables originally consisting of multiple categories were reduced to two levels. In Model 1, HCWs were classified, according to questions B1a and B1c, as those who knew the risk for a HCW of acquiring both HCV and HIV infections from a patient versus all others; in Model 2, they were grouped, according to questions B4 and B6, as those who knew that using standard precautions (gloves, mask, protective eyewear) and hands hygiene after removing gloves are HAIs control measures versus all others; and in Model 4, HCWs were grouped, according to questions D3 and D5, to whether they often or always used gloves when at direct contact with a patient and hands hygiene measures after removing gloves versus all others. The following independent variables were included in all models: gender (male = 0, female = 1), age (continuous, in years), working category (physician = 0, nurse = 1), number of years in practice (continuous), number of patients seen in a workday (continuous), number of working hours in a week (continuous), knowledge about the risk for a HCW of transmitting HCV and HIV infections to a patient (no = 0, yes = 1), knowledge that HCV and HIV infections can be serious (no = 0, yes = 1), knowledge that HCWs' hands are vehicle for transmission of nosocomial pathogens (no = 0, yes = 1), educational courses and scientific journals as sources of information about HAIs (no = 0, yes = 1), and need of additional information about HAIs (no = 0, yes = 1). The following variables were also included: knowledge that the use of standard precautions is a HAIs control measure (no = 0, yes = 1) in Model 1; knowledge about the risk for a HCW of acquiring HCV and HIV infections from a patient (no = 0, yes = 1), and knowledge that invasive procedures are a risk factor for HAIs (no = 0, yes = 1) in Models 2-4; marital status (single/separated/divorced/widowed = 0, married = 1), number of other persons in the household (0 = 0, 1 = 1, 2 = 2, 3 = 3, 4 = 4, >4 = 5), and knowledge that using standard precautions (gloves, mask, protective eyewear) and hands hygiene after removing gloves are HAIs control measures (no = 0, yes = 1) in Model 3; knowledge that hands hygiene after removing gloves is a HAIs control measure (no = 0, yes = 1), positive attitude towards the use of guidelines for HAIs control practices (no = 0, yes = 1), positive attitude toward hands hygiene measures to reduce the risk among patients (no = 0, yes = 1), positive attitude toward hands hygiene measures to reduce the risk among HCWs (no = 0, yes = 1), and perceived risk of acquiring a HAI (continuous) in Model 4.

The primary analysis was univariate and the variables significantly associated with the outcomes of interest at *p*-value of 0.25 or less were included in the final models. Then, one stepwise multivariate linear regression model and three multivariate logistic regression models were constructed and the significance level for the variables to entry in the model was set at 0.2 and for removal at 0.4. In the logistic models the association between predictors and outcomes was measured by odds ratios (ORs) and their 95% confidence intervals (CIs). All tests were two-tailed and a *p*-value of 0.05 or less was defined as statistically significant. The data were analyzed using the statistical software Stata [14].

## Results

Of the 550 surveys distributed, a total of 307 participants returned the questionnaire with a final response rate of 55.8%. Two-thirds of the respondents were male, the mean age was 44 years, the mean number of years in practice was 11, and the mean number of patients seen in a day was 30.

Answers concerning the knowledge of HCWs are reported in Table 1. A majority (87.9%) were aware that a HCW can acquire HCV and HIV from a patient, but less than one-third knew that a HCW can transmit these infections to a patient. Table 2 shows the results of the multivariate analysis regarding the association between the different outcomes of interest and the various explanatory variables. HCWs with fewer number of years of practice (OR = 0.9; 95% CI 0.85-0.96), who worked fewer hours in a week (OR = 0.9; 95% CI 0.84-0.97), who knew the risk for a HCW of transmitting HCV and HIV infections to a patient (OR = 6.07; 95% CI 1.31-28.14), who knew that HCV and HIV infections can be serious (OR = 8.09; 95% CI 3.31-19.81), who have received information about HAIs from educational courses and scientific journals (OR = 3.54; 95% CI 1.22-10.24), and who did not need additional information about HAIs (OR = 0.06; 95% CI 0.01-0.55) were more likely to know the risk for a HCW of acquiring both HCV and HIV from a patient (Model 1). The vast majority correctly identified as proper HAIs control measures the use of gloves, mask, and protective eyewear (94.1%) and hands hygiene measures after removing gloves (91.5%). Overall, 86.3% were aware of both preventive measures and this knowledge was significantly higher in nurses (OR = 2.34; 95% CI 1.09-5.01), in HCWs who provided care to fewer patients in a day (OR = 0.98; 95% CI 0.95-0.99), who knew that HCWs' hands are vehicle for transmission of nosocomial pathogens (OR = 4.64; 95% CI 1.85-11.68), who received information about HAIs from educational courses and scientific journals (OR = 3.54; 95% CI 1.47-8.5), and who did not know the risk for a HCW of transmitting HCV and HIV infections to a patient (OR = 0.24; 95% CI 0.11-0.5) (Model 2).

**Table 1:** Knowledge about health care-associated infections and control measures

Number of question	Questions (correct response)	n	%
*Health care-associated infections that a healthcare worker can acquire from a patient*			
B1b	Hepatitis C (true)	289	94.1
B1c	Human Immunodeficiency Virus (true)	277	90.2
B1h	Tetanus (false)	264	86
B1d	Influenza (true)	189	61.6
B1a	Hepatitis B (true)	177	57.7
B1i	Tuberculosis (true)	122	39.7
B1f	Mumps (true)	43	14
B1g	Rubella (true)	43	14
B1l	Varicella (true)	40	13
B1e	Measles (true)	35	11.4
*Health care-associated infections that a healthcare worker can transmit to a patient*			
B2h	Tetanus (false)	297	96.7
B2d	Influenza (true)	210	68.4
B2b	Hepatitis C (true)	95	30.9
B2c	Human Immunodeficiency Virus (true)	74	24.1
B2i	Tuberculosis (true)	48	15.6
B2a	Hepatitis B (true)	45	14.7
B2f	Mumps (true)	17	5.5
B2l	Varicella (true)	16	5.2
B2e	Measles (true)	14	4.6
B2g	Rubella (true)	14	4.6
*Control measures*			
B6	Wearing gloves, mask, and protective eyewear (true)	289	94.1
B4	Hands hygiene measures after removing gloves (true)	281	91.5
B5	Changing mask before going to another patient (true)	222	72.3
*Risk factors*			
B7	Invasive procedures (true)	281	91.5
B8	HCWs' hands are vehicle for transmission of nosocomial pathogens (true)	275	89.6

HCW = Healthcare worker

**Table 2:** Multivariate logistic (1, 2, 4) and linear (3) regression models results

Variable	OR	95% CI	*p*
**Model 1. HCWs who know the risk of acquiring HCV and HIV infections from a patient**			

Log likelihood = -81.13, χ^2 ^= 63.68 (8 df), *p *< 0.0001			
Know that HCV and HIV infections can be serious	8.09	3.31-19.81	<0.001
Fewer number of years in practice	0.9	0.85-0.96	0.002
Fewer number of working hours in a week	0.9	0.84-0.97	0.006
Need of additional information about HAIs	0.06	0.01-0.55	0.012
Educational courses and scientific journals as sources of information about HAIs	3.54	1.22-10.24	0.02
Know the risk for a HCW of transmitting HCV and HIV infections to a patient	6.07	1.31-28.14	0.021
Older age	1.06	0.99-1.14	0.08
Fewer number of patients seen in a day	0.99	0.96-1.01	0.32

**Model 2. HCWs who know that using standard precautions and hands hygiene after removing gloves are HAI's control measures**			

Log likelihood = -98.84, χ^2 ^= 47.37 (6 df), *p *< 0.0001			
Not know the risk for a HCW of transmitting HCV and HIV infections to a patient	0.24	0.11-0.5	<0.001
Know that HCWs hands are vehicle for transmission of nosocomial pathogens	4.64	1.85-11.68	0.001
Educational courses and scientific journals as sources of information about HAIs	3.54	1.47-8.5	0.005
Working as a nurse	2.34	1.09-5.01	0.029
Fewer number of patients seen in a workday	0.98	0.95-0.99	0.05
Fewer number of years in practice	0.97	0.93-1.01	0.16

**Model 4. HCWs who often or always use gloves when at direct contact with a patient and performed hands hygiene measures after removing gloves**			

Log likelihood = -114.73, χ^2 ^= 71.02 (10 df), *p *< 0.0001			
Know that hands hygiene after removing gloves is a HAIs control measure	8.09	2.83-23.1	<0.001
Fewer number of patients seen in a workday	0.97	0.95-0.99	0.014
Working as a nurse	2.33	1.13-4.79	0.022
Know that invasive procedures are a risk factor for HAI	2.69	0.92-7.84	0.07
Educational courses and scientific journals as sources of information about HAIs	2.15	0.89-5.2	0.09
Know the risk for a HCW of acquiring HCV and HIV infections from a patient	2.22	0.88-5.58	0.09
Higher perceived risk for a HCW of acquiring a HAI	1.15	0.96-1.37	0.12
Beliefs that the use of guidelines for HAIs control practices do not reduce the risk	0.4	0.1-1.61	0.2
Not know the risk for a HCW of transmitting HCV and HIV infections to a patient	0.62	0.27-1.4	0.25
Younger age	0.98	0.94-1.02	0.31

**Variable**	**Coeff**.	**t**	***p***

**Model 3. HCWs who perceive a risk of acquiring a HAI from a patient**			

F(10,296) = 4.88, *p *< 0.0001, R^2 ^= 14.2%, adjusted R^2 ^= 11.3%			
Need of additional information about HAIs	1.23	3.86	<0.001
Working as a nurse	0.66	3.01	0.003
Educational courses and scientific journals as sources of information about HAIs	0.76	2.43	0.016
Know that HCWs hands are vehicle for transmission of nosocomial pathogens	0.72	1.98	0.049
Know the risk for a HCW of transmitting HCV and HIV infections to a patient	0.32	1.24	0.22
Fewer number of years in practice	-0.02	-1.2	0.23
Know that HCV and HIV infections can be serious	0.37	1.19	0.23
Know the risk for a HCW of acquiring HCV and HIV infections from a patient	0.38	1.06	0.29
Higher number of other persons in the household	0.07	1.03	0.3
Higher number of patients seen in a workday	0.01	0.98	0.33
Constant	3.6		

HCW = Healthcare worker; HCV = Hepatitis C Virus; HIV = Human Immunodeficiency Virus; HAI = Health care-associated infection

Concerning the perceived risk of acquiring a HAI, HCWs' thought to be at high risk with a mean value of 7.3. The multivariate linear regression analysis showed that being a nurse, knowing that HCWs' hands are vehicle for transmission of nosocomial pathogens, obtaining information about HAIs from educational courses and scientific journals, and needing additional information about HAIs were significantly independently associated with a higher level of perceived risk (Model 3). Moreover, HCWs had an extremely positive attitudes since 94.5% and 89.2% agreed that guidelines for preventing HAIs should strictly be followed and that hands hygiene measures after treating patients reduces the risk, respectively.

Answers concerning the HCWs who often or always adopt practices to reduce the risk of HAIs are reported in Table 3. Only 57.3% always wore gloves and 85.2% of them reported always changing gloves after each patient, while 52.3% and 79% always performed hands hygiene measures before and after wearing gloves, respectively. A total of 80.8% of respondents often or always used gloves and performed hands hygiene measures after removing gloves. This behavior was more frequent in nurses (OR = 2.33; 95% CI 1.13-4.79), in HCWs who provided care to fewer patients (OR = 0.97; 95% CI 0.95-0.99), and who knew that hands hygiene after removing gloves was a control measure (OR = 8.09; 95% CI 2.83-23.1) (Model 4).

**Table 3:** Healthcare workers who often or always adopt practice to reduce the risk of health care-associated infections

Number of question	Practice	n	%
D10	Placing needles in sharp's containers	278	90.5
D3	Wearing gloves when at direct contact with a patient	272	88.6
D6	Changing gloves before going to another patient	267	87
D2	Hands hygiene measures before going to another patient	266	86.6
D5	Hands hygiene measures after removing gloves	264	86
D1	Hands hygiene measures before starting the working activity	240	78.2
D4	Hands hygiene measures before wearing gloves	202	65.8
D9	Recapping needles after using	151	49.2
D7	Wearing protective eyewear when at direct contact with a patient	110	35.8
D8	Wearing mask when at direct contact with a patient	109	35.5

The most commonly reported source of information about HAIs was educational courses (71%) followed by scientific journals (48.2%); 85.3%, however, claimed to need to update what they already knew.

## Discussion

In this present investigation, a questionnaire was utilized to collect information from a sample of HCWs in randomly selected emergency care setting of Italian hospitals regarding their knowledge, attitudes, and behaviors about HAIs.

Participants' knowledge concerning the various aspects of HAIs was generally high and consistent with current scientific evidence, since the vast majority were aware about some infections that a HCW can acquire from a patient and the standard precautions. In contrast, there are wide areas where the knowledge was lower, particularly regarding infections that a HCW can transmit to a patient. Based on this consideration, this specific population needs to learn more in order to reduce the rate of HAIs. Continuing medical benefits in the hospital environment require continuing educational input.

In this investigation, the working activity was found to be a significant determinant of the amount of knowledge about standard precautions and hands hygiene after removing gloves as control measures for HAIs, their perceived risk of acquiring a HAI, using gloves and performing hands hygiene measures. Nurses were more likely to have a higher level of knowledge, to have a higher perceived risk, and to use appropriate HAIs' control measures than physicians. It is possible that such differences may be attributed to the more active involvement in preventive activities regarding HAIs. Moreover, provision of information about HAIs influence knowledge and behaviors because HCWs were able to answer correctly and to appropriately use HAIs control measures if they have received information from educational courses and scientific journals. This shows that providing HCWs with appropriate information is enough to ensure understanding, especially in a particular risk group like the sample of this study.

Results from this nationwide survey indicate that most respondents often or always used gloves and performed hands hygiene measures after removing gloves for the prevention of the HAIs. No differences were observed in reported compliance with recommendations according to gender and age of the HCWs. Instead, two independent predictors of compliance were positively associated: fewer patients cared in a day and know that hands hygiene measures after removing gloves is a control measure. The finding that lower knowledge is linked to the underuse of appropriate control measures confirm the need to intensify educational programs. Moreover, the use of protective barriers was considerably lower than those observed in previous surveys. For instance, in a sample of emergency medicine residents in the United States, 96% and 99% used gloves at least 95% of the time for irrigation and incision and for drainage procedures, respectively [11]. Physicians and nurses in pediatric EDs in Canada self-reported a high rate of handwashing before and after all patients with a mean score, out of 5 possible points, of 4.9 and 4.5, respectively, and for wearing gloves when examining patients of 3.3 and 3.2 [10]. A national telephone survey among orthopedic surgeons in accident and EDs throughout England found that 99% routinely used gloves in a major trauma scenario, but only 18% and 21% used face mask and eye protection, respectively [12]. Finally, our values were higher than those in EDs in the United Kingdom and New Zealand, with values of 27% and 58% and of 14% and 12%, respectively, for asepsis in invasive procedures and hands hygiene between patient consultations [9].

Another key finding was that the attitudes towards HAIs are encouraging, since a high percentage of respondents reported positive global and specific beliefs. In particular, 94.5% indicated that guidelines should be established and followed. The multivariate analysis indicated that being nurses, knowing that HCWs' hands are vehicle for transmission of nosocomial pathogens, requiring and receiving information about HAIs were significantly independent predictors of a high perceived risk of acquiring a HAI.

There are some potential limitations in the design and measurements of this study that should be considered when interpreting the results. First, it provides as a cross-sectional study, only circumstantial evidence for the casual nature of the relationships that have been observed. No direct relationship between variables and outcomes can be proved but substantial evidence has been demonstrated for the association discussed. A second limitation is the potential reporting bias associated with the self-administered questionnaire. Concern always exists about accuracy in these surveys and it is difficult to determine with certainty whether the responses reflect what HCWs actually do. Specifically, compliance to control measures was based solely upon the subjective views of HCWs with the possibility that they tend to over-report compliance, notwithstanding that all interviews were anonymous. A more effective method of measuring compliance would be the direct observation of actual practice although the effect of being monitored may improve compliance by itself. A final limitation was that the response rate of 55% was disappointingly low, and one reason may be the time constraints faced by busy practitioners. We were not able to gather detailed information on non-respondents and, therefore, we were unable to assess whether there was a subgroup that systematically failed to respond. Although this response rate does not reflect internal validity of the findings, it may decrease the overall generalizability of the results to all HCWs in EDs. However, because HCWs tend to be relatively homogeneous with respect to attitudes and behaviors, the response rate may not have led to significant non-response bias.

## Conclusions

HCWs in EDs show high levels of knowledge and positive attitudes, but the low compliance rate with regard to standard precautions about HAIs clearly reveal the urgency to implement initiatives for improving healthcare policies and to stress the need for adopting and following preventive recommendations by all HCWs.

## Competing interests

The authors declare that they have no competing interests.

## Authors' contributions

CP participated in the design of the study, statistical analysis, interpretation of the data, and collected the data. RA participated in the statistical analysis and interpretation of the data. PM participated in the design of the study and interpretation of the data. IFA, the principal investigator, designed the study, was responsible for the data collection, statistical analysis and interpretation of the data, and wrote the article. All Authors read and approved the final manuscript.

## APPENDIX A

Tables 4, 5, 6, 7 and 8.

**Table 4:** Demographic and occupational characteristics section of the questionnaire

A. DEMOGRAPHIC AND OCCUPATIONAL CHARACTERISTICS
This section is designed to gather information about your socio-demographic and occupational characteristics.
**A1**. What is your gender? □ Male □ Female
**A2**. How old were you on your last birthday? _____ years
**A3**. What is your marital status? □ Married □ Single (never married) □ Other (**specify **____)
**A4**. How many persons are there in your household? (not counting you) _____
**A5**. What is your working category in the Emergency Department (ED)? ____
**A6**. How many years have you been working in an ED? ____
**A7**. How many patients do you provide care in a day in the ED? _____
**A8**. How many hours per week do you work in the ED? _____

**Table 5:** Knowledges section of the questionnaire

B. KNOWLEDGES			
This section is designed to explore your knowledge related to health care-associated infections (HAIs)			
**B1**. Which of the following infections a healthcare worker (HCW) can acquire from a patient? **(check one or more)**			
**a**.□ Hepatitis B	**b**.□ Hepatitis C	**c**.□ Human Immunodeficiency Virus	**d**.□ Influenza
**e**.□ Measles	**f**.□ Mumps	**g**.□ Rubella	**h**.□ Tetanus
**i**.□ Tuberculosis	**l**.□ Varicella		
**B2**. Which of the following infections a HCW can transmit to a patient? **(check one or more)**			
**a**.□ Hepatitis B	**b**.□ Hepatitis C	**c**.□ Human Immunodeficiency Virus	**d**.□ Influenza
**e**.□ Measles	**f**.□ Mumps	**g**.□ Rubella	**h**.□ Tetanus
**i**.□ Tuberculosis	**l**.□ Varicella		
**B3**. Which of the following infections can be serious? **(check one or more)**			
**a**.□ Hepatitis B	**b**.□ Hepatitis C	**c**.□ Human Immunodeficiency Virus	**d**.□ Influenza
**e**.□ Measles	**f**.□ Mumps	**g**.□ Rubella	**h**.□ Tetanus
**i**.□ Tuberculosis	**l**.□ Varicella		
For each statement regarding HAIs, please check whether you agree, are uncertain or disagree			
	**Agree**	**Uncertain**	**Disagree**
**B4**. Hands hygiene after removing gloves is a HAIs control measure	□	□	□
**B5**. Changing mask before going to another patient is a HAIs control measure	□	□	□
**B6**. Wearing gloves, mask, and protective eyewear are a HAIs control measures	□	□	□
**B7**. Invasive procedures are a risk factor for HAIs	□	□	□
**B8**. HCWs' hands are a vehicle for transmission of nosocomial pathogens	□	□	□

**Table 6:** Attitudes section of the questionnaire

C. ATTITUDES									
This section is designed to explore your attitudes towards HAIs. For each statement check whether you agree, are uncertain or disagree.									
	**Agree**	**Uncertain**	**Disagree**						
**C1**. The use of guidelines for HAIs control practices reduce the risk	□	□	□						
**C2**. Hands hygiene measures reduce the risk of HAIs among patients	□	□	□						
**C3**. Hands hygiene measures reduce the risk of HAIs among HCWs	□	□	□						
**C4**. How do you perceive your risk of acquiring a HAI on a 1 to 10 scale with 1 meaning no risk and 10 very much risk?									
1	2	3	4	5	6	7	8	9	10
No risk									Very much risk

**Table 7:** Behaviors section of the questionnaire

D. BEHAVIORS					
This section is designed to gather information about your behaviors. Check how often do you adopt each of the following practices to reduce the risk of HAIs.					
	**Always**	**Often**	**Sometimes**	**Rarely**	**Never**
**D1**. Hands hygiene measures before starting the working activity	□	□	□	□	□
**D2**. Hands hygiene measures before going to another patient	□	□	□	□	□
**D3**. Wearing gloves when at direct contact with a patient	□	□	□	□	□
**D4**. Hands hygiene measures before wearing gloves	□	□	□	□	□
**D5**. Hands hygiene measures after removing gloves	□	□	□	□	□
**D6**. Changing gloves before going to another patient	□	□	□	□	□
**D7**. Wearing protective eyewear when at direct contact with a patient	□	□	□	□	□
**D8**. Wearing mask when at direct contact with a patient	□	□	□	□	□
**D9**. Recapping needles after using	□	□	□	□	□
**D10**. Placing needles in sharp's containers	□	□	□	□	□
**D11**. Using syringes with retractable needle	□	□	□	□	□
**D12**. Using syringes with protective shield	□	□	□	□	□
**D13**. Using scalpels with protective shield	□	□	□	□	□
**D14**. Using intravenous cannulation with retractable needle	□	□	□	□	□

**Table 8:** Source of information section of the questionnaire

E. INFORMATION			
This section is designed to ask questions about your sources of information.			
**E1**. From which of the following sources do you receive information about HAIs? **(check one or more)**			
□ None	□ Scientific journals	□ Mass-media	□ Educational courses
□ Physicians	□ Other **(please specify________)**		
**E2**. Do you feel you need more information about HAIs?		□ Yes	□ No

## Pre-publication history

The pre-publication history for this paper can be accessed here:

http://www.biomedcentral.com/1471-2334/10/35/prepub
